# Advanced microscopy analysis of the micro-nanoscale architecture of human menisci

**DOI:** 10.1038/s41598-019-55243-2

**Published:** 2019-12-10

**Authors:** V. Vetri, K. Dragnevski, M. Tkaczyk, M. Zingales, G. Marchiori, N. F. Lopomo, S. Zaffagnini, A. Bondi, J. A. Kennedy, D. W. Murray, O. Barrera

**Affiliations:** 1 Università degli Studi di Palermo, Palermo, Italy; 2 University of Oxford, Oxford, UK; 3 IRCCS Istituto Ortopedico Rizzoli, Laboratorio di Biomeccanica e Innovazione Tecnologica, Bologna, Italy; 4 Università degli Studi of Brescia, Brescia, Italy; 5 University of Luxembourg, Luxembourg, Luxembourg; 6 Oxford Brookes University, Oxford, UK

**Keywords:** Biophysics, Anatomy

## Abstract

The complex inhomogeneous architecture of the human meniscal tissue at the micro and nano scale in the absence of artefacts introduced by sample treatments has not yet been fully revealed. The knowledge of the internal structure organization is essential to understand the mechanical functionality of the meniscus and its relationship with the tissue’s complex structure. In this work, we investigated human meniscal tissue structure using up-to-date non-invasive imaging techniques, based on multiphoton fluorescence and quantitative second harmonic generation microscopy complemented with Environmental Scanning Electron Microscopy measurements. Observations on 50 meniscal samples extracted from 6 human menisci (3 lateral and 3 medial) revealed fundamental features of structural morphology and allowed us to quantitatively describe the 3D organisation of elastin and collagen fibres bundles. 3D regular waves of collagen bundles are arranged in “honeycomb-like” cells that are comprised of pores surrounded by the collagen and elastin network at the micro-scale. This type of arrangement propagates from macro to the nanoscale.

## Introduction

**Figure 1:**
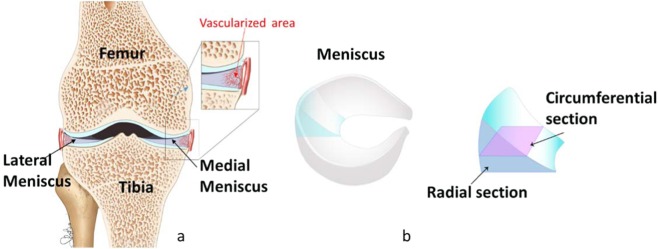
(**a**) Schematic representation of the right knee showing the position of the menisci. (**b**) Schematic representation of the lateral meniscus’ shape and the direction of the radial (blue) and circumferential (purple) sections.

The meniscus is a tough fibro-cartilage porous soft tissue that conforms to the surfaces of the bones upon which it rests within the knee joint. There are two menisci: lateral and medial, each one is located between the femur and tibia (Fig. 1a). The meniscus has evolved to fulfil a specific role within the knee i.e. load transmission to the articular cartilage, structural stability and impact absorption^1–7^. Recently the impact absorption function has been debated^7^. The natural evolution of tissues and organs fulfilling specific roles can be seen as perfect examples of multi-objective optimization for achieving enhanced performance in the face of constraints, which are mainly energetic^8^. For example, improved mechanical strength requires more nutrients, hence vascularization of the tissue is enhanced. In contrast, the need of improved mechanical endurance - essential in the case of sustaining repetitive and cyclic mechanical loading as in the case of the meniscus - does not require vascularization to the same extent. Only 10% to 25% of a healthy meniscus periphery is vascularized. The loss of the meniscus leads to joint degeneration and osteoarthritis as contact stresses on the tibial plateau increase proportionally with the amount of meniscus tissue removed^9^. Hence, replacing the resected meniscal tissue by an artificial implant might be necessary in order to avoid the articular cartilage degeneration. Currently, the clinical and functional outcomes for these devices are not ideal as unable to replicate the function of the native meniscus^10^. The understanding of structure-function relationship of the meniscus among other soft porous tissues is the key to make a breakthrough in the development of biological implants to repair, maintain or improve tissue functions; however, it remains a significant challenge. One of the main issue leading to the lack of this understanding is related to the difficulty of identifying and quantifying architectural features that are crucial for the functionality of the tissue.

The meniscus is composed of Extracellular Matrix (ECM) whose main constituents are fluid and porous solid phases. The fluid phase is mainly water (60–70%) while the solid phase consists primarily of collagen type I (15–25%) interposed with cells^11^. Proteoglycans (PGs), glycoproteins and non-collagenous proteins account for the remaining dry weight. To-date studies on the structure-function of the meniscal tissue conclude that collagen fibers running along the circumferential direction (Fig. 1b) are the main feature ruling the load bearing capacity of the meniscus. A pioneer study conducted at the macroscopic level with polarised light microscopy on fixed meniscal tissues highlighted the link between tensile strength and orientation of collagen fibers^12^. Results showed that (a) the collagen fibres in the meniscus are mainly arranged circumferentially to withstand the tension and (b) the fibres oriented radially act as tie fibres to avoid “longitudinal splitting of the meniscus”. The study in^12^ has opened the way for further studies of the collagen fibers organization at different length scales, with other more advanced techniques available at the time, which overall confirmed the findings. Over two decades ago Scanning Electron Microscopy (SEM) measurements on human meniscus samples revealed for the first time the 2D structure and orientation of the collagen fibers^13^. Recently, other light and electron microscopic and optical projection tomography studies elucidated the complex structure of the knee menisci^14,15^. In particular, in^14^ the authors highlighted new insight on the arrangement of the circumferential and radial (Fig. 1b) section of manipulated (i.e. fixed, dehydrated) human meniscal samples. Collagen fibrils in the radial section (radial tie fibers) are observed to be arranged into 10 μm bundles, which “associate laterally to form flat sheets of varying dimensions that bifurcate and come together to form a honeycomb network within the body of the meniscus”^14^. Each of these honeycomb compartments is filled with collagen fibrils (diameter of 5 μm) that are tightly packed together to form the collagen array running in the circumferential direction^14^. Despite the important and innovative findings reported in these papers, it is important to note that their results are limited by the technique and more precisely by the associated preparation of the samples, i.e. include fixation, mechanical and chemical peeling, dehydration and metallization among others. These procedures alter to a greater or lesser extent the tissue structure limiting the quantitative aspect of the results^16^. Therefore, systematic studies using up-to-date non-invasive microscopy methods such as confocal Multi-Photon (MP) fluorescence/second-harmonic generation microscopy and Environmental Scanning Electron Microscopy (ESEM) and have proved to be a valuable method to analyse the architecture of living tissues-without the need for tissue processing. MP microscopy enables accurate microscale 3D reconstruction of the main structural proteins of living tissues i.e. collagen and elastin^16–28^. Despite the difficulty in distinguishing the collagen and elastin signals, ESEM is suitable as allows 2D imaging of tissue structure down to the nanoscale^29,30^.

Microscale structure of bones, tendons, cardiovascular tissue, and cartilage were studied by MP microscopy and detailed information about architecture and orientation of elastin and collagen bundles were highlighted^16–28^. MP studies on bovine meniscus, for example, have shown that in circumferential sections a high density of tightly packed collagen bundles is present, characterised by noticeably larger diameter (~100 μm). SHG vertical section image reveals the arboreal organization of the radial fibers that “tie together” the meniscus^25^. SHG studies of the radial section of the bovine meniscus show the 3D arrangement of the radial tie fibers dividing the larger fascicles into smaller bundles. Among tissues intrinsic fluorescence, it is possible to distinguish elastin signal. Indeed, elastin auto fluorescence is usually brighter than other endogenous signal due to its peculiar supramolecular arrangement in filaments^18–22^. Here we present an experimental study coupling detailed information coming from Environmental Scanning Electron Microscopy (ESEM) and MP spectroscopy, which allows reconstructing without any sample treatment, of collagen and elastin fibers architecture of the human meniscal tissue. We were able to define and quantify regular wave-like arrangement of collagen bundles both at micro and nano scale in the circumferential section. In the radial section, we observed collaged bundles waves organised in such a way that they seem to divide the cross section of the meniscal tissue in compartments of honeycomb-like shape as reported in^14^. Interestingly, we observe that each of these honeycomb compartments is not filled with collagen fibrils running in the circumferential direction as noted in^15^. Moreover, we were able to observe that these honeycomb-like compartments propagate from macro to nano-scale. The degree of packing of the honeycomb-like compartments and the porosity play certainly a determinant role in the poro-mechanics behaviour of the meniscal tissue.

## Result and Discussion

We show in this section details of meniscal architecture at micro-nano scale. In particular, we will refer to the two types of section showed in Fig. 1b, i.e. circumferential and radial sections. While measurements performed in the circumferential direction overall agree with the current literature, they are novel finding as they provide quantitative information regarding (a) collagen bundle orientations, (b) size of gaps between bundle sheets, (c) periodicity and wavelength of collagen bundles, (d) elastin fibers thickness and orientation with respect collagen bundles. On the other hand, our findings related to the radial section are novel and show that collagen bundles form the side walls of micro-nano highly porous honeycomb-like cell structure which develop in space as micro channels.

**Figure 2:**
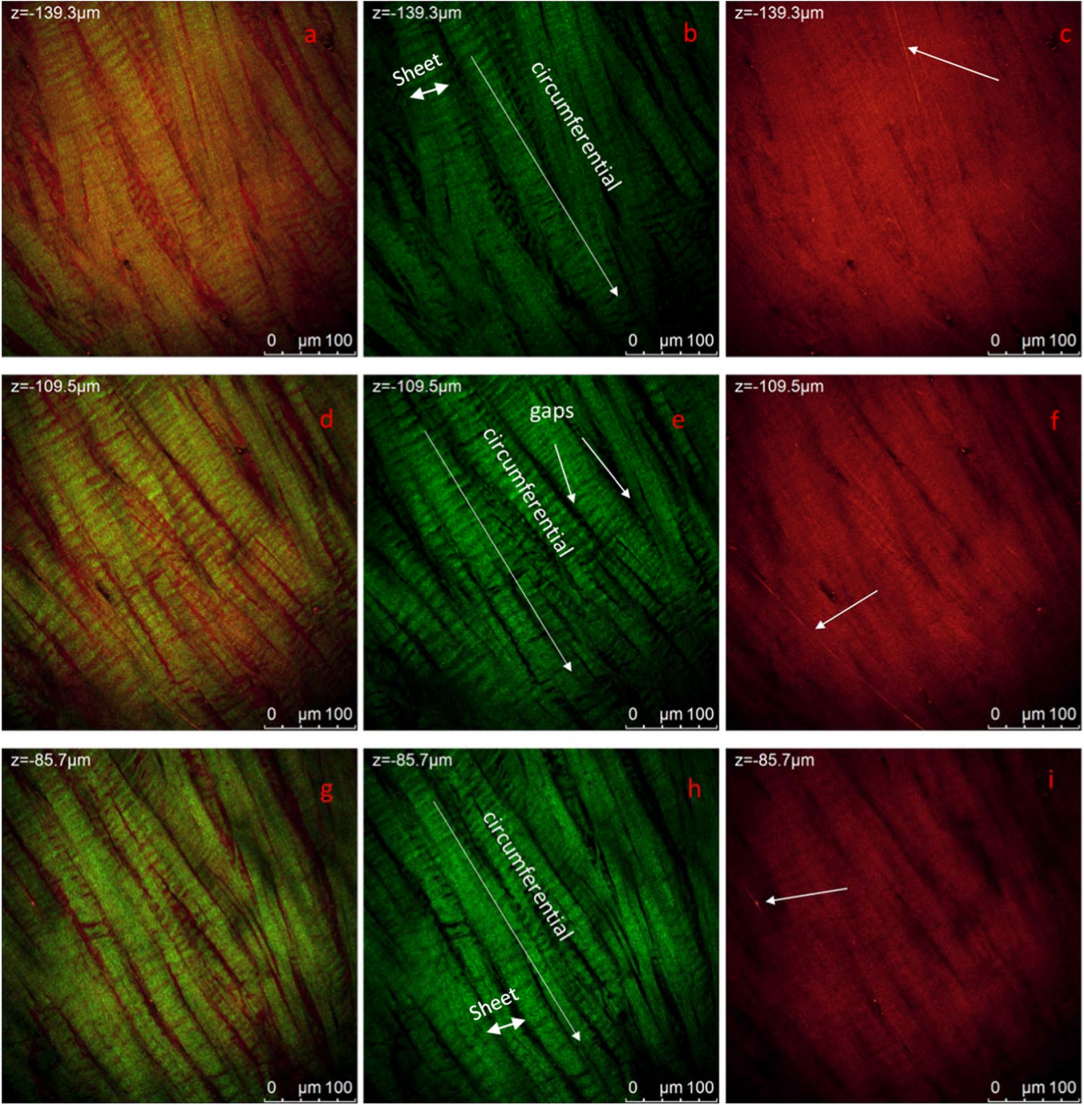
Circumferential section of the central portion of the lateral meniscus. Representative 1024 × 1024 section of Z-Stacks at different heights are reported. The thickness of the explored sample is 60 μm, measurements are acquired with 3um steps under MP laser excitation at λ_exc_ = 880 nm, SHG signal is acquired in the range 390–460 nm (green channel) and tissue autofluorescence in the range 485–650 nm. In panel (a,d,g) the overlay of the two channels is reported for measurements at 3 different representative heights while in the other panels the same measurements in separated spectral ranges are reported.

**Figure 3:**
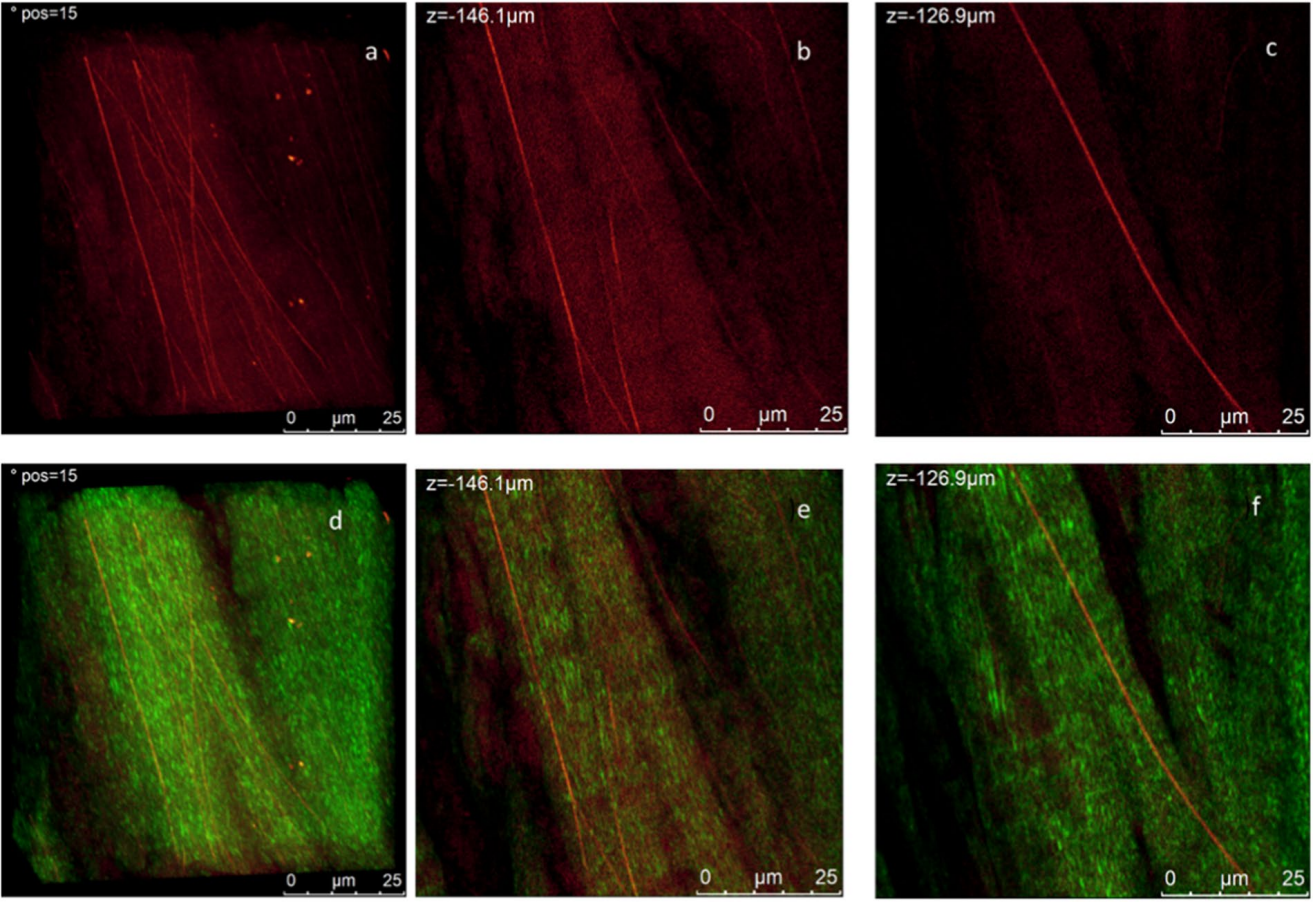
Circumferential section of the central portion of the lateral meniscus. 3D reconstruction of the autofluorescence/SHG signal of a higher magnification of Fig. 2. Elastin fibers are clearly visible as filaments of about 1 μm thickness. Representative 1024 × 1024 section of Z-Stacks at different heights are reported. Measurements are acquired with 3 μm steps under MP laser excitation at λexc = 880 nm, SHG signal is acquired in the range 390–460 nm (green channel) and tissue autofluorescence in the range 485–650 nm. (**a**) 3D projection of measurement in the red channel (**b**,**c**) wo representative sections at different heights. (**d**) 3D projection of the SHG and autofluorescence signal overlapped (**e**,**f**) same section in panel b and c where SHG signal is overlapped Scale bar is 10 μm and step size is about 1 μm. The video collecting images of complete measurements are reported in SI.

Images of the internal circumferential section of untreated human meniscus were acquired along the z axis with a 3 μm step for 60 μm. In Fig. 2a–g we show three representative 387.5 μm × 387.5 μm sections at different heights. Images were acquired using 880 nm laser excitation allowing simultaneously revealing SHG signal (green, Fig. 2b,e,h) due to collagen fibrils as SHG signal around 430 nm. Fluorescence signal (red, Fig. 2c,f,i) acquired in the range 485 nm–650 nm is due to tissues autofluorescence: diffused background can be due to several components in the tissue such as Proteoglycans (PGs), glycoproteins and non-collagenous proteins^19^. Elastin fibrils are detectable as thin fibrils with higher intensity of fluorescence signal^19^. These are visible in Fig. 2c,f,i. A higher resolution measurement on the same area at higher magnification is presented in Fig. 3. A 3D reconstruction of images acquired in the red channel showing elastin fibers is observed in Fig. 3a. Video is available in Supporting Information (Video_Fig. 3).

Figure 3b,c shows the arrangement of elastin fibers in two representative sections at different heights. It can be noted that the elastin fibers appear to follow the same orientation of the collagen bundles as shown in Fig. 3d–f where the SHG and auto fluorescence signals are overlapped. It was possible to approximately estimate the thickness of a single elastin fiber to be around 700 nm-1 μm.

**Figure 4:**
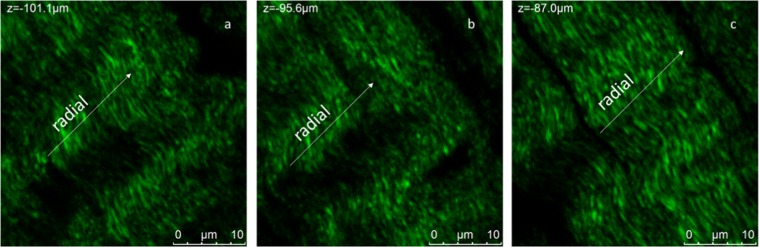
Circumferential section of the central portion of the lateral meniscus. Representative 1024 × 1024 section of Z-Stacks at different heights are reported in (**a**–**c**). Measurements are acquired with 500 nm steps under MP laser excitation at λ_exc_ = 880 nm, SHG signal is acquired in the range 390–460 nm (green channel). Weave bundles of collagen fibrils with a diameter of about 600 nm in XY plane are aligned in the circumferential direction.

The observation of the previous images reveals a regular organisation of collagen at different scales. As evident in Fig. 2, collagen bundles (b, e, h-green channel) appear to be arranged in 30–50 μm sheets (as highlighted in Fig. 2b,h) with the majority of sheets being aligned with a specific orientation along the circumferential direction, indicated by arrows in Fig. 2b,e,h. It is worth noting that the collagen sheets are separated by gaps (black background in the green channel) of variable dimension ranging from few to tens of micrometres, which might be associated to voids through which fluid can flow or other material which does not give SHG. SHG signal intensity presents maxima and minima with a repeatable pattern along the sheets axis which represents circumferential direction. At this length scale collagen appears in shape of waves aligned in this direction. In Fig. 4 we report representative images of the same region of the sample in Fig. 2 at increased magnification, this allows to revealing structural organisation of collagen at higher resolution. Only images acquired in the green channel reporting SHG signal attributed to collagen is shown.

At this length scale, bundles of collagen fibrils with a diameter of about 600 nm are aligned in the circumferential direction. Each bundle is constituted by a high number of thin collagen fibrils which appears twisted and tangled up together. These bundles are in shape of continuous waves whose peaks are aligned in a direction orthogonal to the circumferential direction (radial direction). It is interesting to note how the spacing between the collagen sheets evolves thought the thickness of the sample. In this case, at the base of the sample (Fig. 4a) the sheets appear to be connected to each other. At about 5 μm the collagen fascicles begin to separate (Fig. 4b), a clear gap of about 1 μm occurs at about 10 μm depth from the base (Fig. 4c).

It is important to better quantify orientation and periodicity of the collagen bundles in order to link this information with the mechanical properties of the tissue and build appropriate models to predict the behaviour of the tissue under loading. Fourier analysis has been already combined with SHG microscopy, with the aim of quantifying collagen fibers organization in a range of porcine tissues such as porcine tendons^31^ and cornea trachea and ear cartilage^32^. We quantified collagen bundle orientation as the perpendicular direction to the one were FFT maxima aligns^33,34^.

**Figure 5:**
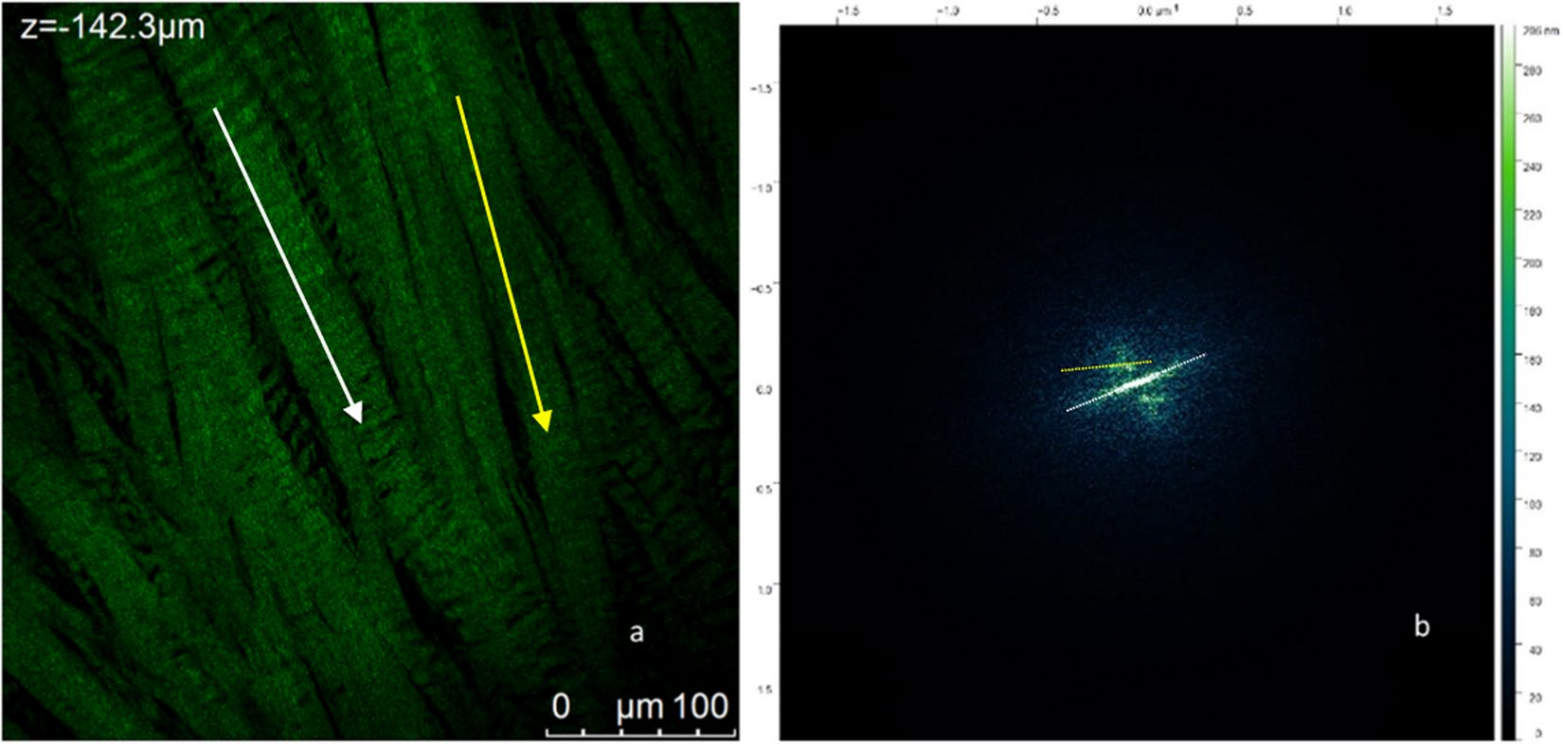
(**a**) One example of Z-stack of the circumferential section of the central portion of the lateral meniscus as shown in Fig. 2 white and yellow arrows correspond to preferential fibres orientation as obtained by FFT analysis. (**b**) FFT of image in (**a**) characterized by two narrow lobes revealing a period structure along two main directions which are indicated by white and yellow dashed lines. Details on the FFT analysis are given in Supporting Information (SI, FFT analysis.docx).

A representative 387.5 × 387.5 μm SHG image of the human meniscus sample is reported in Fig. 5a) together with the corresponding 2D Fourier transform of the entire image in Fig. 5b).

It can be observed that the FFT presents two narrow lobes corresponding to two highly regularly oriented populations of weavy/crimped collagen bundles indicated by white and yellow lines in panel b). One preferential orientation is found to be oriented at 110° with respect to the vertical axis, the other at 100° which correspond to preferential orientations at 20° and 10° respectively (corresponding white and yellow arrows in panel a). It is evident that the crimps/waves are fairly regular in both the populations of collagen bundles, hence it is also possible to analyse the spatial frequency of these two populations by extracting a line spectrum from the 2D FFT along the preferential directions and quantifying the distance between two symmetric maxima.

We performed the FFT analysis on analogous measurements in this sample which gave an average wavelength of collagen waves of about 15 μm in both directions. Hence, we were able to determine two populations of collagen sheets oriented at 20° and 10° degrees with respect the vertical with the same characteristic wavelength of 15 μm.

**Figure 6:**
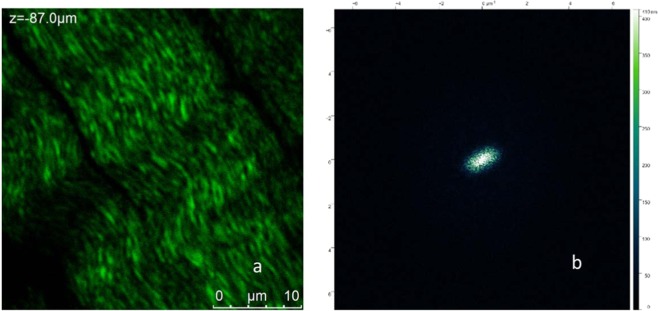
(**a**) One example of Z-stack of a high magnification of the circumferential section of the central portion of the lateral meniscus as shown in Fig. 4. (**b**) FFT of image in (**a**) with one wider lobe revealing a limited periodicity at this scale.

It is possible to extend this analysis on lower space scale to obtain a more detailed quantification of the regular wave arrangement of collagen fibril bundles. As in fact, it is evident in Fig. 4 that shorter wavelength collagen waves, made of nanoscale fibrils, are aligned in the radial direction. For this purpose, we report in Fig. 6a representative image of meniscus at 10 μm scale and in Fig. 6b the correspondent FFT analysis. In contrast with Fig. 5b, in which there were evident narrow lobes, at this scale a single broad lobe is present whose “average” orientation is 11° degrees from the vertical which is consistent to what seen in Fig. 5b. FFT analysis on a number of analogous Z-stacks of 33 µm (Fig. 6a) shown in Fig. 6b allows the estimation of a characteristic wavelength for collagen bundles of about 1.8 µm. ESEM measurements were performed on analogous samples to confirm the findings using a completely different method and improve image resolution to the scale of tens nanometres.

**Figure 7:**
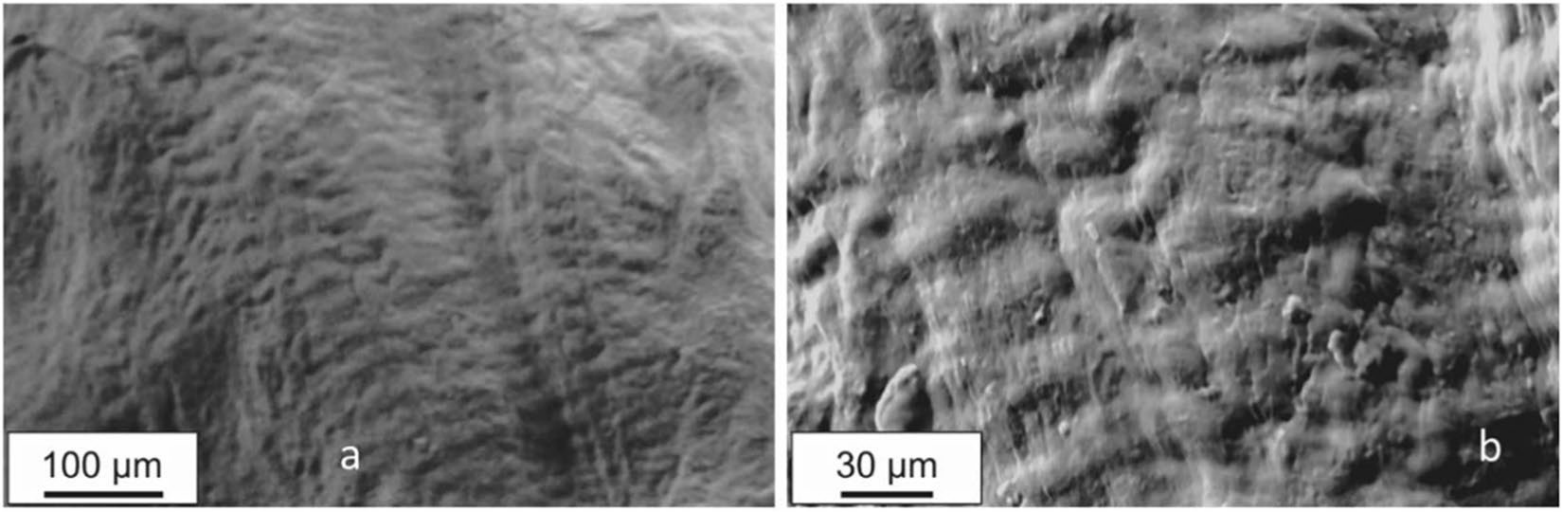
Lateral meniscus, posterior region. (**a**) ESEM image of uncoated specimens of the circumferential section obtained at a pressure of 10Pa, scale bar 100 µm. (**b**) Higher magnification of an area of (**a**), scale bar 30 µm.

In Fig. 7a ESEM measurements on a sample cut along the circumferential on a similar scale to the one presented in Fig. 2 is presented. Analogous features are observed to the one highlighted by MP Microscopy. In Fig. 7b a representative measurement on 10 µm scale is reported. From both images the roughness of the surface resembles the collagen wave morphology with analogous characteristic features. Measurements in Fig. 7a does not reveal different features than the ones attributable to collagen. In Fig. 7b small details are evident, i.e. sub micrometric roundish structures and thin ridges apparently filamentous that have same size and spatial organisation of elastin. Despite the fact that these measurements do not add new information, they support previous observations. A close correlation with what observed by means MP microscopy is observed at this scale, despite the diversity of samples origin.

**Figure 8:**
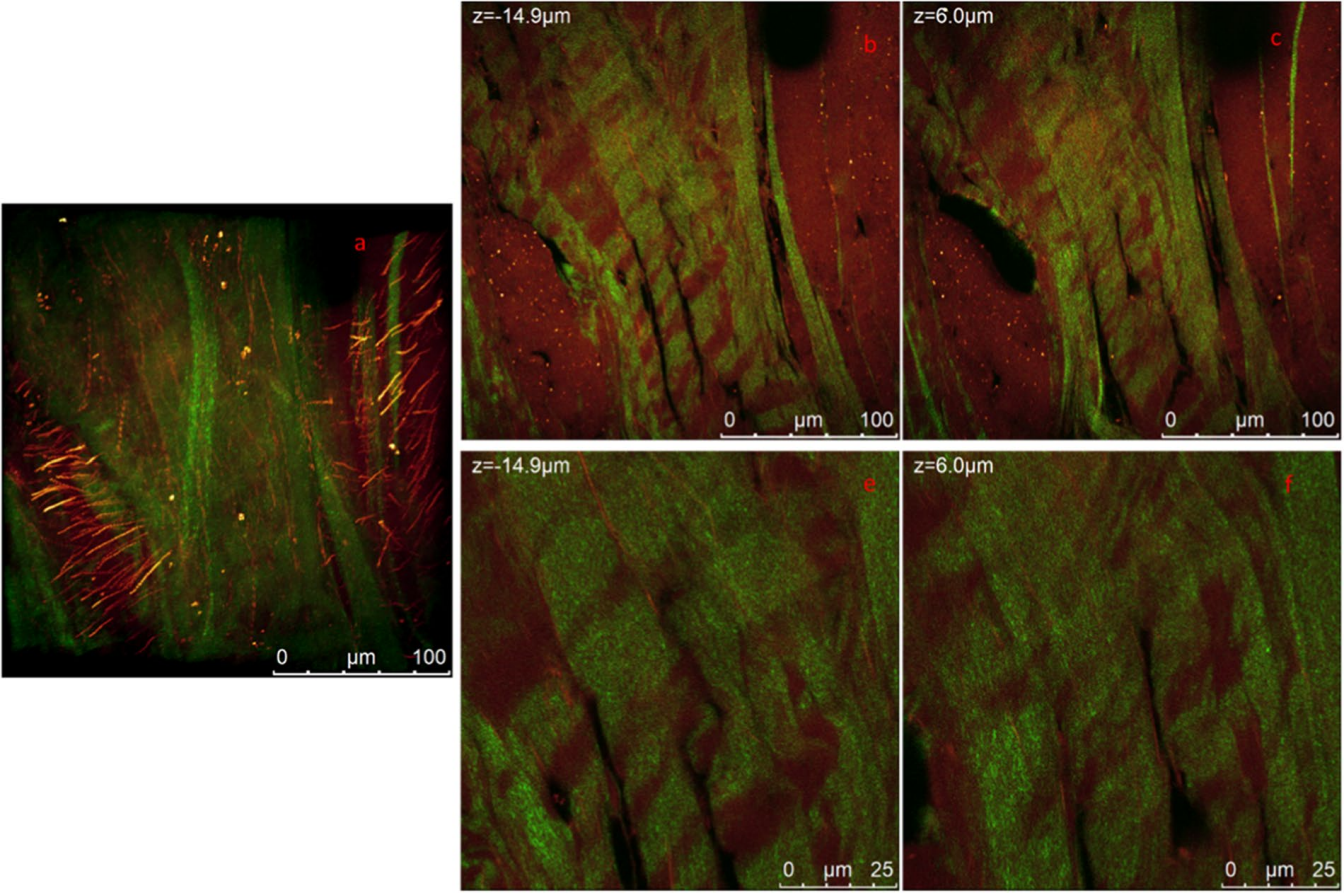
MP analysis of the radial section of the central portion of the lateral meniscus. (**a**) 3D reconstruction of a 60 um depth measurement. The overlap of SHG (green channel) and autofluorescence (red channel) is reported. (**b**,**c**) Show two representative 1024 × 1024 images from the Z-stack at different heights. (**e**,**f**) Higher resolution images from an analogous z stack acquired at higher magnification of the same region shown in (**b**,**c**). Measurements are acquired under MP laser excitation at λ_exc_ = 880 nm, SHG signal is acquired in the range 390–460 nm (green channel) and tissue autofluorescence in the range 485–650 nm. The video collecting images of complete measurements are reported in SI.

In Fig. 8 we report multiphoton analysis of a sample cut along the radial section. In panel a) we report a representative 3D reconstruction of the radial section of untreated human meniscus see SI for video related to the measurements; images were acquired along the z axis for 60 µm depth. Representative sections at different heights are shown in panel b) and c), again SHG signal and autofluorescence signal are reported in green and red respectively. In Fig. 8e,f higher magnification measurements in the same region are shown in Fig. 8b,c.

The structural complexity of meniscus architecture is immediately evident. The homogeneous and regular organization observed previously in the circumferential section is not observed. Collagen sheets, whose width ranges between 5 and 10 µm, run along the image. Thick and long elastin filaments are more evident than what has been observed in the circumferential section. In the 3D reconstruction presented in Fig. 8a it is possible to appreciate a group of collagen bundles arranged in straight and weavy patterns which are enclosed by elastin fibers oriented along a direction perpendicular to the collagen bundles/sheets. In Fig. 8e,f it has been noted that there might be a possible link between the arrangement and orientation of the collagen bundles and elastin fibers. At this stage, elastin fibers appear to have the same orientation of the collagen bundles. If compared to radial section, collagen in the circumferential section appear to be arranged in a less compact structure organized in a helicoidally way. Video in SI (video_Fig. 8a,b,d) better highlights the cylindrical symmetry of this portion of the sample.

**Figure 9:**
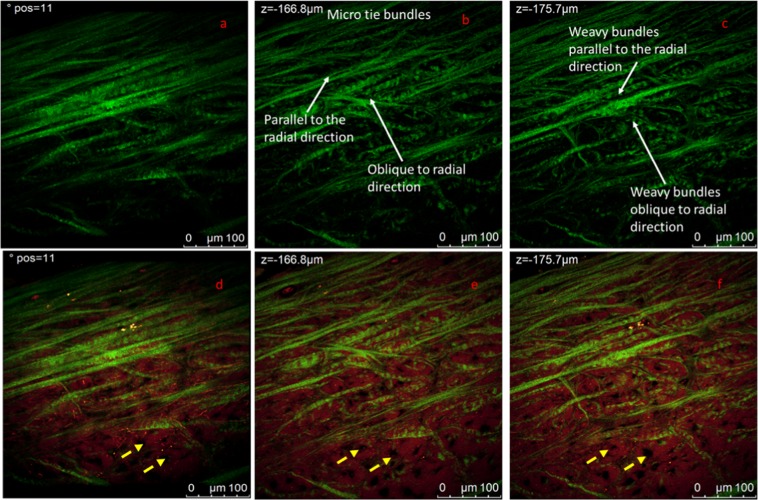
(**a**) 3D reconstruction of the SHG signal of the radial section of the posterior region of the lateral meniscus. (**b**,**c**) Representative 1024 x1024 section of Z-Stacks at different heights. Collagen bundles in shape of waves and straight bundles are observed. (**d**) 3D projection of the SHG and autofluorescence signal overlapped. (**e**,**f**) same section in panel b and c where autofluorescence signal is overlapped. The straight micro tie-fibers-bundles appear to divide the radial section in micro compartments more visible in the zoom in Fig. 10. Yellow arrows indicate the presence of black round holes which might be associated with pores or non-fluorescence material. Measurements are acquired with 3 um steps under MP laser excitation at λ_exc_ = 880 nm, SHG signal is acquired in the range 390–460 nm (green channel) and tissue autofluorescence in the range 485–650 nm. The video collecting images of complete measurements are reported in SI.

**Figure 10:**
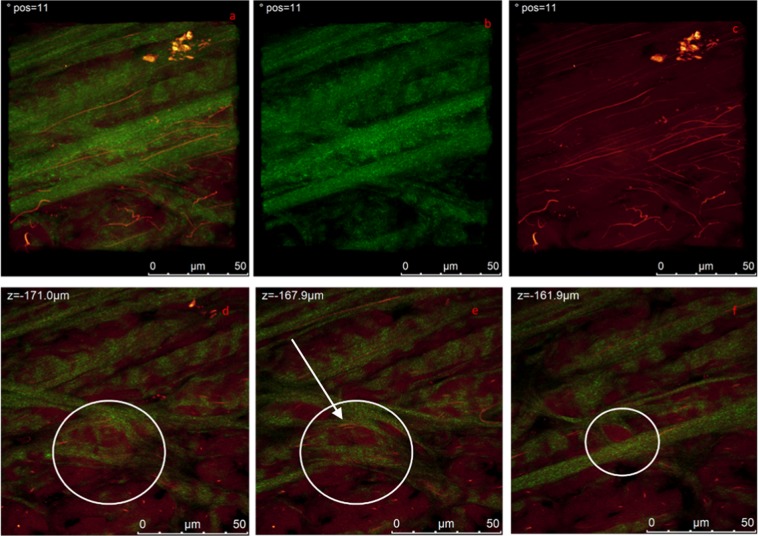
(**a**,**c**) Shows a 3D reconstruction of a higher magnification of the same region shown in Fig. 9. (**a**) Combined SHG and autofluorescence signals, (**b**) SHG signal (collagen), (**c**) autofluorescence signal (elastin) revealing similar arrangement and orientation of collagen bundles and elastin fibers. (**d**–**f**) Present the details of three Z-stacks showing a small micro-compartment of about 25 µm width (white circles).

A 3D reconstruction of images of the radial section of the posterior region acquired in the green channel, where collagen bundles are evident, is observed in Fig. 9a. Video is available in Fig. 9. (a) 3D reconstruction of the SHG signal of the radial section of the posterior region of the lateral meniscus. (b,c) Representative 1024 × 1024 section of Z-Stacks at different heights. Collagen bundles in shape of waves and straight bundles are observed. (d) 3D projection of the SHG and autofluorescence signal overlapped. (e,f) same section in panel b and c where autofluorescence signal is overlapped. The straight micro tie-fibers-bundles appear to divide the radial section in micro compartments more visible in the zoom in Fig. 10. Yellow arrows indicate the presence of black round holes which might be associated with pores or non-fluorescence material. Measurements are acquired with 3um steps under MP laser excitation at λexc = 880 nm, SHG signal is acquired in the range 390–460 nm (green channel) and tissue autofluorescence in the range 485–650 nm. The video collecting images of complete measurements are reported in SI.

Supporting Information (Video_Fig. 9). Figure 9b,c shows 1024 × 1024 images representing the arrangement of collagen bundles in two representative sections at different heights. It can be noted that collagen bundles in the green channel are not widely distributed. Long and straight fibrils of about 5 µm width go through the whole image and FFT analysis of analogous measurements in this sample revealed information on the orientation of these fibrils to be at −10° and 18° with respect of the horizontal axis. Another family of bundles is characterised by wave shape bundles. Measurements were acquired on 387.5 μm × 387.5 μm region along the z axis with a 3 μm step for 60 μm. Figure 9d–f report the SHG and auto fluorescence signals overlapped. These straight micro-tie-bundles, highlighted in panel b, run both parallel or obliquely to the radial direction, appearing arranged in such a way that might divide the region into a series of micro compartments similarly to what reported in^14^. The size of each compartment can vary between 25–75 µm, this is more evident in the SI. This more evident in higher magnification in Fig. 10d–f. Moreover, the shape of these compartments are more noticeable from ESEM measurement as discussed later in this section. In Fig. 9 in the red channel the uniform autofluorescence signal presents circular holes of 30–40 μm diameter highlighted by yellow arrows. This may represent empty space as well as region where non fluorescent material is present. Interestingly, weavy bundles appear to follow the same direction as the straight ones, as highlighted in panel c).

Figure 10a–c shows a 3D reconstruction of a higher magnification of the same region shown in Fig. 9. At this scale it was possible to reveal insight on the possible correlation between the collagen bundles and elastin fibers organisation.

Collagen structure appear to have at least two preferential directions which is closely followed by elastin fibrils. Video can be found in SI (video_Fig. 10). Figure 10d–f presents the details of three Z-stacks, it is interesting to picture a small micro-compartment of about 25 µm width. White arrow in panel e is guideline to the eye. This small micro-compartments have been also observed to be inside larger compartments in analogous measurements (data not reported). Inside each compartment at the microscale we did not observe circumferentially oriented collagen bundles as seen by other authors at the same scale^13,14,24^.

**Figure 11:**
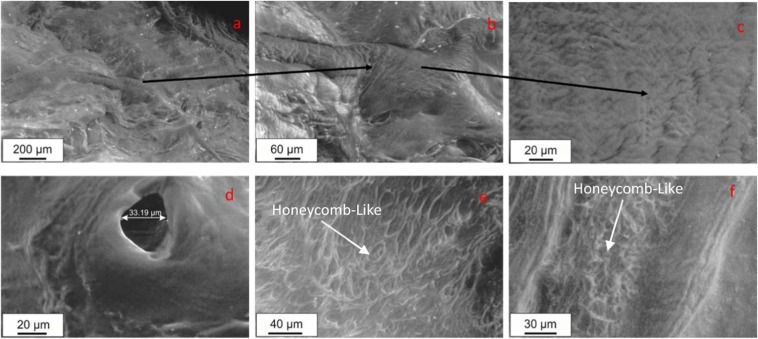
Lateral meniscus, posterior region. ESEM image of uncoated specimens of the radial section obtained at a pressure of 10Pa. (**a**) Three of these large tie fibers (60–80 μm width) more similar to bundle sheets (yellow lines) emanating/conveying into a common “node” (red circle). (**b**) higher magnification on the node in (**a**). (**c**) Details of the roughness of the large tie bundle sheets. (**d**) A high resolution image of pore of about 30 μm. (**e**) Microscale view of the portion inside a macro honeycomb compartment. (**f**) Micro-honeycomb- like structure is delimited by micro tie fiber bundles with a diameter of about 5 μm highlighting tie fiber bundles and a “honeycomb-like” network. ESEM images of hydrated meniscus specimens (P = 533Pa, T = −5 °C).

Large views of the radial section have been examined by optical microscopy (ESEM) and have been compared with same observation in^15^. Observations show that (apparently) straight large tie fibers bundle sheets (80 μm width) run in radial and oblique direction creating an intricate network which “tie” and also divide the meniscal tissue into a series of macro compartments of which may resemble a Honeycomb-Like (H-L) structure with dimension of about 0.1–0.6 mm. ESEM results of details of the radial section are reported in Fig. 11. Figure 11a shows a representative view of the radial section at the macroscale. It is possible to note three of these large tie fibers (60–80 μm width) bundle sheets emanating/conveying into a common “node”. A zoom of this “node” is reported in Fig. 11b,c in which these tie bundles appear constituted by crimped fibrils running parallel and obliquely to the direction of the tie bundles. The meniscus at this length scale presents several pores as the one circular shaped with a diameter of about 30 microns shown in Fig. 11d. Size and morphology of this circular hole are comparable with MP observations in Fig. 9. ESEM observations at higher magnification scale in Fig. 11e,f revealed that the H-L is a recurrent self-similar type of structure from the macroscale to the microscale.

A higher magnification of a region inside one of the macro-honeycomb compartment is shown in Fig. 11e in which tie fiber bundle sheets of decreasing size (10–20 μm width) appear running radially through the section with an inter tie fiber region characterized by a H-L network at the microscale. The micro H-L structure is delimited by fiber bundles with a diameter of about 5 μm and with a distance between two corners of about 10–50 µm.

A zoom of this region can be visualized in Fig. 11f.

The morphology of the honeycomb-like region is comparable to the MP observations reported in Fig. 9. ESEM appears to be a more powerful tool to reveal this kind of structure which is certainly evident also from MP analysis but here is more clear and shown with more details.

## Conclusion

In this paper we have investigated the organization of the human meniscal tissue using up-to-date non-invasive imaging techniques, which allow minimal treatment of the sample, thus minimising artefacts or tissue distortions due to staining or fixation procedure. The described experimental procedure, based on Multi Photon fluorescence and second harmonic generation microscopy supplemented by Environmental Scanning Electron Microscopy measurements coupled with quantification methods such as Fast Fourier Transform (FFT), has enabled highlighting three-dimensional organization of the collagen and elastin matrices. The analysed samples come both from intact menisci (ESEM) and from patients undergoing total knee arthroplasty (MP microscopy), this being a limitation of this study. However, we note that analogous features are evident for both conditions at the analysed spatial scale and overall the presented study reveals the potential of the combination of advanced microscopy techniques and quantitative methods in unveiling architectural features never observed before. Our findings are summarized below:

- We were able to define and quantify regular wave-like arrangement of collagen bundles both at micro and nano scale in the circumferential section of human meniscal samples. This overall agrees with the literature (on porcine samples), however for the first time 3D details and quantification are discussed in the present study.
- In the radial section we observed collaged bundles waves organised in such a way that they divide the cross section of the meniscal tissue in compartments of honeycomb-like shape. This feature has been already reported in^14,15^. However, our findings differ from^14^ as we observe that each of these honeycomb compartments is not filled with collagen fibrils running in the circumferential direction^35,36^.
- We were able to observe that these honeycomb-like compartments propagate from macro to nano-scale.

**Figure 12:**
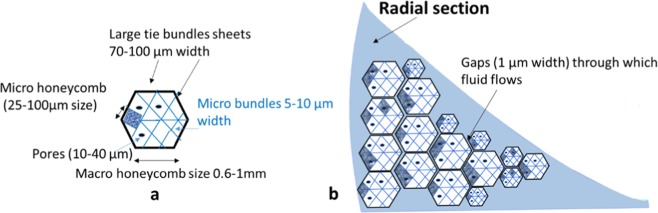
(**a**) Schematic representation of the radial section of the meniscus. Large tie collagen bundles arranged in sheets of 70–100 μm width divide the section into macro polygons of honeycomb-like shape (0.6–1 mm). Each polygon is then divided into micro compartment (25–100 μm) of roughly the same honeycomb shape by collagen bundles of about 5 μm width. Inside these micro compartments there are pores of mostly circular shape with about 10–40 μm of diameter, fluid flows through these pores. (**b**) The whole radial section is formed by a number of macro honeycomb shape compartments which are not highly packed. Gaps of about 1–20 μm between each macro compartment are observed which also are believed to allow fluid to flow through.

In order to clarify our findings, we propose a picture of the cross section (schematically represented in Fig. 12). Our results show the organization in regular waves of collagen bundles arranged in a “honeycomb-like” fashion from macro to nanoscale. Large tie collagen bundles arranged in sheets of 70–100 μm width divide the section into macro polygons of honeycomb-like shape (0.6–1 mm). Micro-tie fibers of about 5 μm width then divide each macro-polygon into micro compartments (25–100 μm) of roughly the same honeycomb shape. Inside these micro compartments there are pores of mostly circular shape with diameters of about 10–40 μm, fluid flows through these pores (highlighted by ESEM measurements) contributing to the time-dependent behaviour of this tissue. The whole radial section is formed by a number of honeycomb shape compartments; these macro compartments are not highly packed. Gaps of about 1–20 μm between each macro compartment are observed which also are believed to allow fluid to flow through. This work leads the way to further studies on the detailed architecture of portions of the meniscal tissue as well as on more statistically significant findings.

The potential of the combination of MP, ESEM and FFT can be exploited in deeper and more detailed studies aimed at revealing specific features of both healthy and degraded tissues also considering that the architectural features of the meniscus are location-dependent, therefore differences are expected between different portions of the meniscus and also between vascular and avascular regions.

This work forms a solid base for elucidating the correlation between the nano-microscale structure and mechanical properties of the meniscal tissue. This is an essential step towards building accurate multiscale modelling of the tissue and exploring a range of synthetic materials that could mimic the structure/function characteristic of the meniscal tissue.

## Materials and Methods

### Samples

Observations were performed on 50 meniscal samples (30 used for Multi-Photon microscopy and 20 for ESEM) extracted from 6 human menisci (3 lateral and 3 medial). Two of the six menisci - labelled as healthy by gross investigation of the surgeon - have been retrieved from patients undergoing total knee arthroplasty. Four menisci have been removed from cadaveric knees (bought from Science Care tissue bank) which did not present signs of arthritis.

### Multiphoton microscopy

In order to carry out multiphoton microscopy, human menisci (n = 2, 1 lateral and 1 medial) were harvested from patients (age 65–76, mean 72) undergoing total knee arthroplasty (ethical approval EM 249–2018 21/2017/Sper/IOR EM2, Rizzoli Orthopaedic Institute, Bologna, Italy). All methods were performed in accordance with the relevant guidelines and regulations including informed consent. Samples labelled as degraded by gross investigation of the surgeon were discarded. The menisci were frozen at −80° and defrosted on the day of the MP observation. Medial and lateral menisci were divided into three parts, i.e. anterior, central and posterior. Each part was cryosectioned (Leica CM1900, Germany) in 60 μm slices along the two main directions: radial and circumferential. Samples (n = 30) were mounted on microscope slides with glycerol, sealed and then observed under the multiphoton microscope within a few hours from preparation. Samples were imaged at 1024x1024 pixel resolution using a Leica TCS SP5 laser scanning microscope with a 40x oil objective (Leica Microsystems, Germany) with a scanning frequency of 400 Hz. Images stack were acquired with steps ranging from 0.5 μm to 1.7 μm, along the z axis. The two-photon excitation (Spectra-Physics Mai-Tai Ti:Sa ultra-fast laser) was set at 880 nm. The SHG signal was detected in the range 390–460 nm (green channel) and tissue autofluorescence was detected in the range 485–650 nm (red channel).

### 2D Fast Furier Trasform (FFT)

The Fast Fourier Transform is a convenient image processing tool which is useful in analysing characteristic feature of a spatial domain image. In this process, a 2D image is converted from the spatial domain into the Fourier domain in which it is decomposed into its sine and cosine components. In the Fourier domain, each point represents a particular frequency contained in the spatial domain image. Therefore, it is possible to find the dominant frequencies of the image influencing its geometric structure in the spatial domain.

Gwyddion software (open source) has been used in order to readily perform 2D FFT analysis of microscopy images, hence, to obtain the related Fourier images. In order to confirm results a Matlab (MathWorks) code has been written to perfom FFT analysis. Details of the matlab code and the processing of images are given in Supporting information (SI, FFT analysis.docx).

### Environmental scanning electron microscopy

Human menisci (n = 4, n = 2 lateral and n = 2 medial) were harvested at the Nuffield Orthopaedic Hospital (Oxford, UK) from two knee joints bought from Science Care tissue bank. The knees were frozen at −20° and defrosted on the day of the ESEM observation. Samples (n = 20) were cryosectioned (Bright, model number: OTF5000, London, UK) in 30 μm slices at the Botnar Research Center, NDORMS, University of Oxford, UK and mounted in onto standard aluminium stubs using carbon pads. These were then inserted into the chamber of a Carl Zeiss LS15 VP-SEM (Carl Zeiss Ltd, Cambridge, United Kingdom) equipped with a LaB6 cathode and various detectors for electron imaging. ESEM Imaging was carried out under three different conditions detailed in Supporting information (SI, ESEM conditions.docx).

## Supplementary information

Circumferential section. Collagen bundles and elastin fiber arrangement

Radial section. Collagen bundles and elastin fiber arrangement

Radial section. Details of Collagen bundles and elastin fiber arrangement

Radial section. Additional Details of Collagen bundles and elastin fiber arrangement

Radial Section morphology

Higher magnification of the radial Section morphology

ESEM and FFT conditions
