# Supplementary material for: Advanced microscopy analysis of the micro-nanoscale architecture of human menisci
Source: Sci Rep. 2019 Dec 10;9:18732. doi: 10.1038/s41598-019-55243-2 (PMC6904744; doi:10.1038/s41598-019-55243-2)
Supplement: Supplementary file 7 — ESEM and FFT conditions [file 41598_2019_55243_MOESM7_ESM.pdf]

*Supplementary Information to*

# Advanced microscopy analysis of the micro-nanoscale architecture of human menisci

V. Vetri[1], K. Dragnevski[2], M. Tkaczyk[2], M. Zingales[1], G. Marchiori[3], N. F. Lopomo[4], S. Zaffagnini[3], A. Bondi[3], J. A. Kennedy[2], D. W. Murray[2], O. Barrera[2, 5, 6*]

[1] Università degli Studi of Palermo, Italy; [2] University of Oxford, UK; [3] IRCCS Istituto Ortopedico Rizzoli, Bologna, Italy; [4] Università degli Studi of Brescia, Italy; [5]University of Luxembourg, [6] Oxford Brookes University, UK.

*Corresponding Author: Olga Barrera, olga.barrera@ndorms.ox.ac.uk, NDORMS department, Old Rd, Oxford OX3 7LD, UK

-----------------------------------------------------------------------------------------------------------------

## ESEM IMAGING DETAILS

(1) examination of hydrated samples –specimens were imaged at temperatures and pressures, ensuring 100% humidity was maintained. This was achieved by using a standard Deben Cool Stage and following established protocols detailed elsewhere [31];

(2) examination of partially hydrated specimens –menisci were imaged without any sample preparation at room temperature and air pressures in the region between 10 and 50 Pa, which minimised the detrimental effects of charging during imaging;

(3) examination of dehydrated and gold coated specimens, the thin sections were dehydrated in situ and then gold coated for 60 sec using a Quorum SC6020 Coating machine using a pure gold target and at a current of approximately 18 µm. This was done with the aim of establishing whether coating, on the one hand, affected the structure of the menisci and, on the other hand, whether it

was possible to obtain additional information at higher resolutions, by eliminating the beam skirting effects [32].

All imaging was carried out at an operating voltage of 15kV and working distances in the range of 6 to 8 mm. As above, this ensured no specimen charging and minimal beam damage.

---

## 2D Fast Furier Trasform (FFT)

Matlab code and the processing of 2D FFT images using Gwyddion software

1) Img = **imread** ('image.tif'); Command to import the image, the use of raw square images is recommended. We used 1024x1024 images in .tif format. Img now contain a 2D (1024x1024) matrix.
2) Img = **rgb2gray** (Img); image is converted to grayscale.
3) F = **fft2** (Img); 2D FFT of the grayscale image. F now contains a 2D matrix (1024 x 1024) of Fourier coefficients. Lower frequencies are displayed at the edge of the matrix and higher frequencies in the centre of the matrix.
4) F_shift=**fftshift**(F); the FFT result is shifted in order to display lower frequency at centre of the matrix and the highest frequency at the edge.
5) Fsh_log= **log** (1+abs(Fshift)); logarithmic transformation of the image. This step in necessary as the range of the Fourier coefficients (reflecting the intensity values in the Fourier image) is usually too large to be displayed on the screen, therefore the image appears in black colour.

Most of information in the transform image is given by the low frequencies. The FFT image reveals quantitative information regarding periodic and dominating features of the original image.

In this paper we have obtained quantitative information i.e. collagen waves-bundles orientation and spatial frequency by processing 2D FFT images using Gwyddion software. For this purpose, a line spectrum from the 2D FFT image is extracted along the preferential orientations. These preferential directions are related to the pattern of the original image in space domain, hence, they can be accurately quantified. The result is a graph containing a series of Gaussian/Lorenzian-type of distributions. Spatial frequency i.e. wavelength of the waves-bundles is evaluated by quantifying the distance between two symmetric peaks.
